# Supplementary material for: A broadly-neutralizing antibody against Ebolavirus glycoprotein that potentiates the breadth and neutralization potency of other antibodies
Source: bioRxiv. 2024 Jun 25:2024.06.21.600001. Preprint. [Version 1] doi: 10.1101/2024.06.21.600001 (PMC11230233; doi:10.1101/2024.06.21.600001)
Supplement: Supplement 1 [file media-1.pdf]

Figure S1.

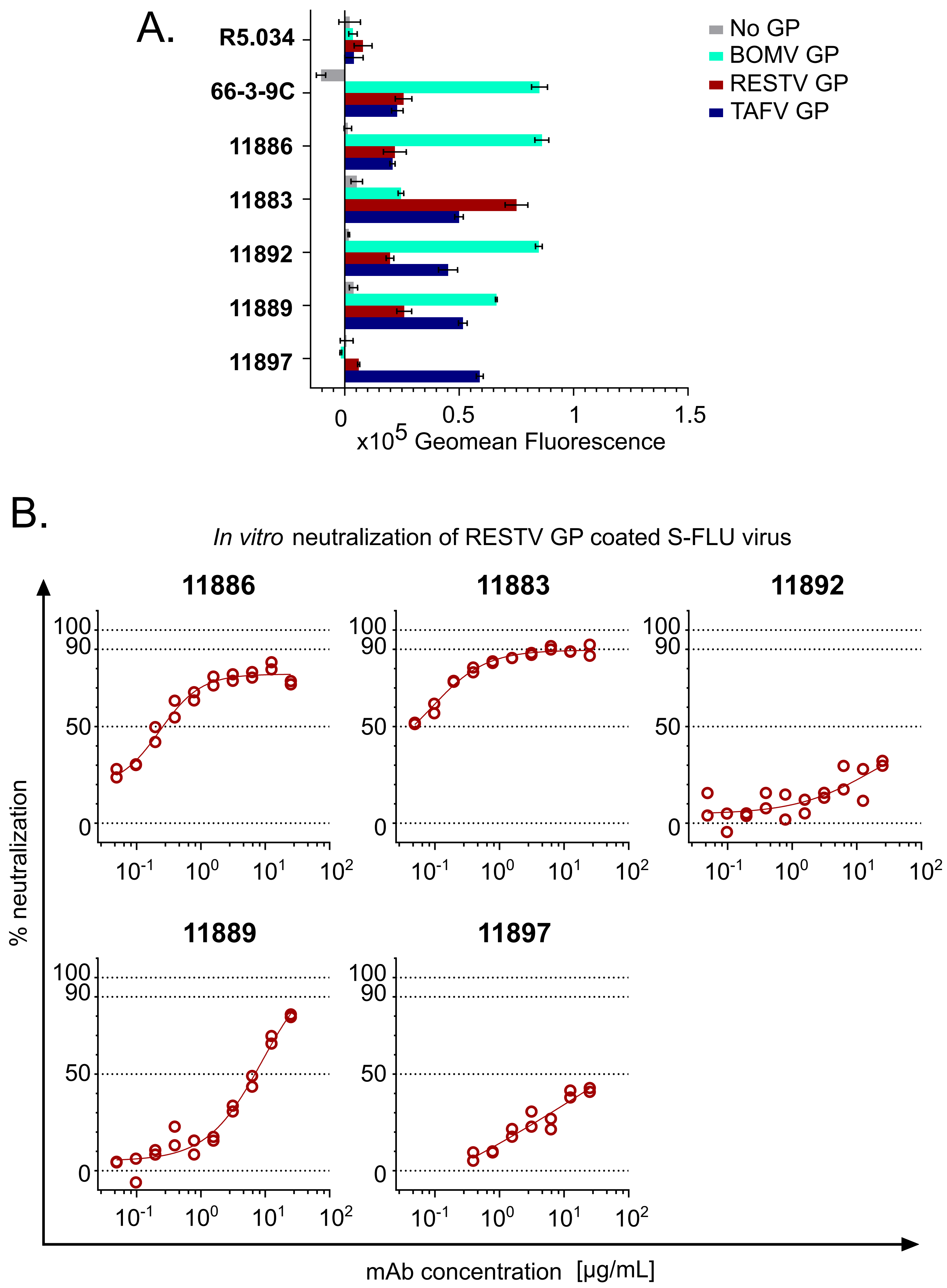
A.
R5.034
66-3-9C
11886
11883
11892
11889
11897
No GP
BOMV GP
RESTV GP
TAFV GP
0
0.5
1
1.5
x10^5 Geomean Fluorescence
B.
In vitro neutralization of RESTV GP coated S-FLU virus
11886
11883
11892
11889
11897
100
90
50
0
10^-1
10^0
10^1
10^2
% neutralization
mAb concentration [µg/mL]

Figure S2.

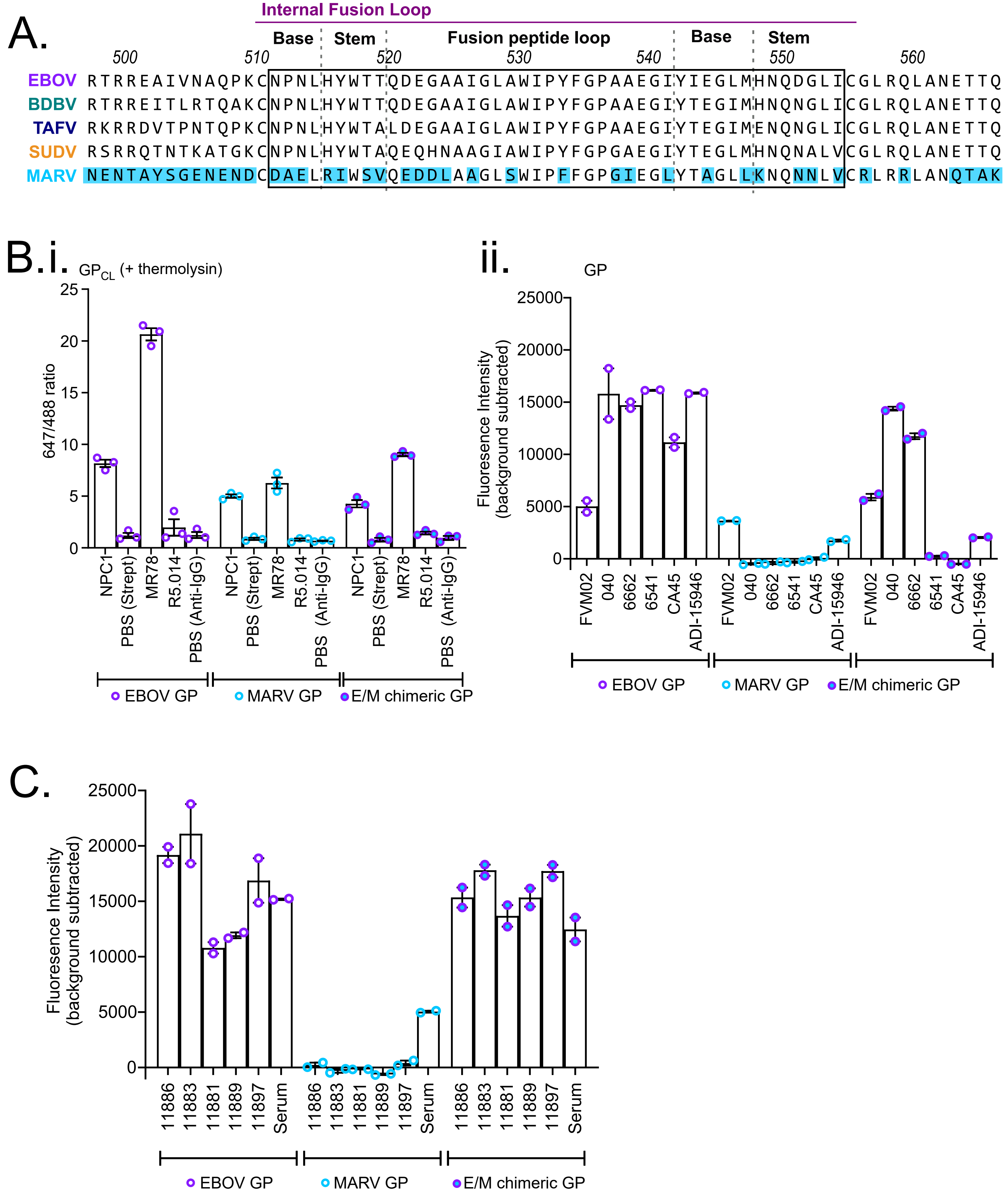

A.
Internal Fusion Loop
Base
Stem
Fusion peptide loop
Base
Stem
500 510 520 530 540 550 560
EBOV RTRREAIVNAQPKCNPNLHYWTTQDEGAAIGLAWIPYFGPAAEGIYIEGLMHNQDGLICGLRQLANETTQ
BDBV RTRREITLRTQAKCNPNLHYWTTQDEGAAIGLAWIPYFGPAAEGIYTEGIMHNQNGLICGLRQLANETTQ
TAFV RKRRDVTPNTQPKCNPNLHYWTALDEGAAIGLAWIPYFGPAAEGIYTEGIMENQNGLICGLRQLANETTQ
SUDV RSRRQTNTKATGKCNPNLHYWTAQEQHNAAGIAWIPYFGPGAEGIYTEGLMHNQNALVCGLRQLANETTQ
MARV NENTAYSGENENDCDAELRIWSVQEDDLAAGLSWIPFFGPGIEGLYTAGLLKNQNNLVCRLRRLANQTAK
B.i. GP_{CL} (+ thermolysin)
647/488 ratio
0 5 10 15 20 25
NPC1
PBS (Strept)
MR78
R5.014
PBS (Anti-IgG)
EBOV GP
MARV GP
E/M chimeric GP
ii.
GP
Fluoresence Intensity (background subtracted)
0 5000 10000 15000 20000 25000
FVM02
040
6662
6541
CA45
ADI-15946
EBOV GP
MARV GP
E/M chimeric GP
C.
Fluoresence Intensity (background subtracted)
0 5000 10000 15000 20000 25000
11886
11883
11881
11889
11897
Serum
EBOV GP
MARV GP
E/M chimeric GP

Figure S3.

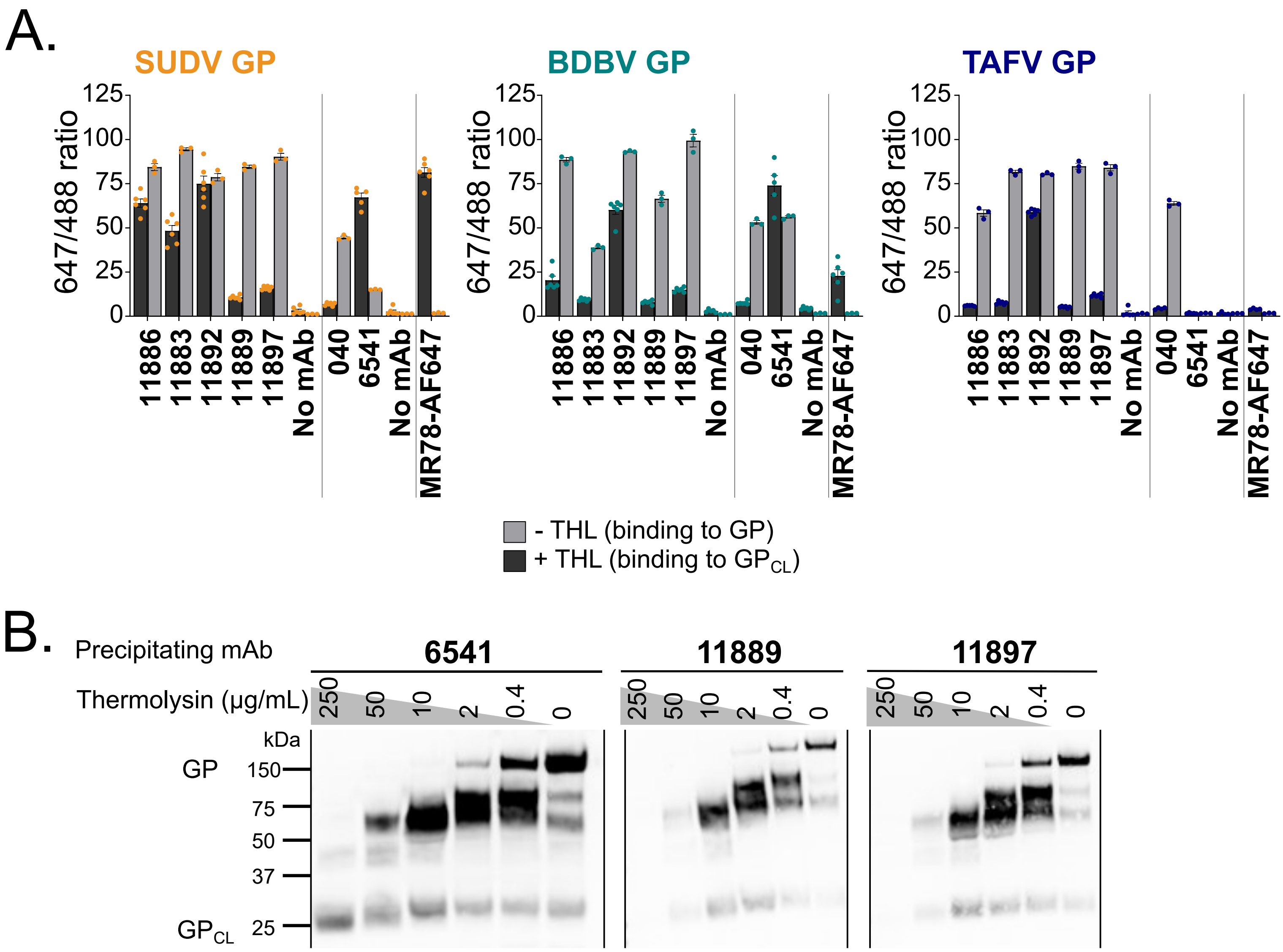

A.
SUDV GP
BDBV GP
TAFV GP
647/488 ratio
125
100
75
50
25
0
11886
11883
11892
11889
11897
No mAb
040
6541
No mAb
MR78-AF647
- THL (binding to GP)
+ THL (binding to GPCL)
B.
Precipitating mAb
6541
11889
11897
Thermolysin (μg/mL)
250
50
10
2
0.4
0
kDa
GP
150
75
50
37
25
GPCL

Figure S4.

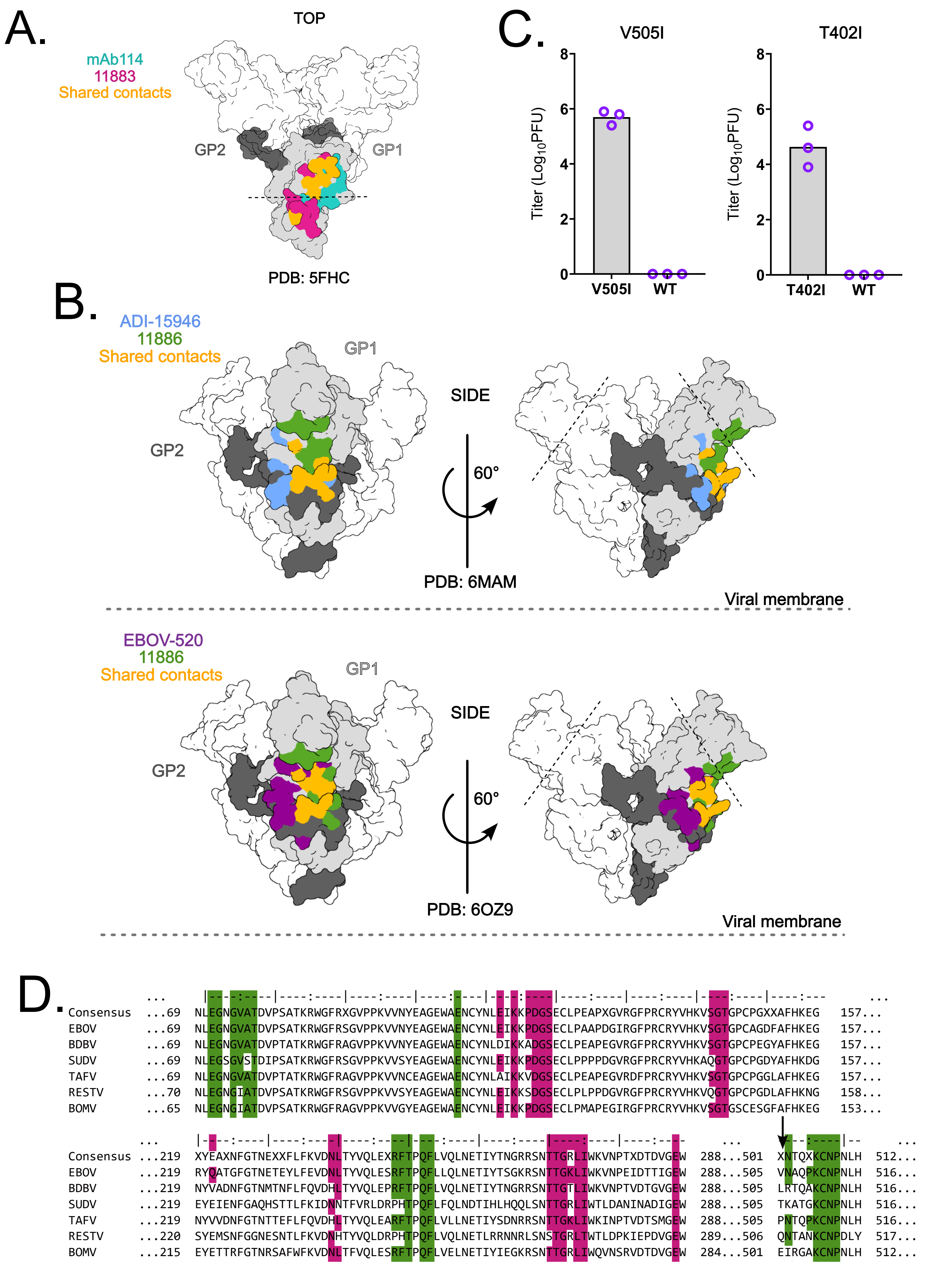
A.
TOP
mAb114
11883
Shared contacts
GP2
GP1
PDB: 5FHC
B.
ADI-15946
11886
Shared contacts
GP1
GP2
SIDE
60°
PDB: 6MAM
Viral membrane
EBOV-520
11886
Shared contacts
GP1
GP2
SIDE
60°
PDB: 6OZ9
Viral membrane
C.
V505I
T402I
Titer (Log10PFU)
0
2
4
6
8
V505I
WT
T402I
WT
D.
Consensus ...69 NLEGNGVATDVPSATKRWGFRXGVPPKVVNYEAGEWAENCYNLEIKKPDGSECLPEAPXGVRGFPRCRYVHKVSGTGPCPGXXAFHKEG 157...
EBOV ...69 NLEGNGVATDVPSATKRWGFRSGVPPKVVNYEAGEWAENCYNLEIKKPDGSECLPAAPDGIRGFPRCRYVHKVSGTGPCAGDFAFHKEG 157...
BDBV ...69 NLEGNGVATDVPTATKRWGFRAGVPPKVVNYEAGEWAENCYNLDIKKADGSECLPEAPEGVRGFPRCRYVHKVSGTGPCPEGYAFHKEG 157...
SUDV ...69 NLEGSGVSTDIPSATKRWGFRSGVPPKVVSYEAGEWAENCYNLEIKKPDGSECLPPPPDGVRGFPRCRYVHKAQGTGPCPGDYAFHKDG 157...
TAFV ...69 NLEGNGVATDVPTATKRWGFRAGVPPKVVNCEAGEWAENCYNLAIKKVDGSECLPEAPEGVRDFPRCRYVHKVSGTGPCPGGLAFHKEG 157...
RESTV ...70 NLEGNGIATDVPSATKRWGFRSGVPPKVVSYEAGEWAENCYNLEIKKSDGSECLPLPPDGVRGFPRCRYVHKVQGTGPCPGDLAFHKNG 158...
BOMV ...65 NLEGNGIATDVPSATKRWGFRAGVPPKVVGYEAGEWAENCYNLEIKKPDGSECLPMAPEGIRGFPRCRYVHKVSGTGSCESGFAFHKEG 153...
Consensus ...219 XYEAXNFGTNEXXFLFKVDNLTYVQLEXRFTPQFLVQLNETIYTNGRRSNTTGRLIWKVNPTXDTDVGEW 288...501 XNTQXKCNPNLH 512...
EBOV ...219 RYQATGFGTNETEYLFEVDNLTYVQLESRFTPQFLLQLNETIYTSGKRSNTTGKLIWKVNPEIDTTIGEW 288...505 VNAQPKCNPNLH 516...
BDBV ...219 NYVADNFGTNMTNFLFQVDHLTYVQLEPRFTPQFLVQLNETIYTNGRRSNTTGTLIWKVNPTVDTGVGEW 288...505 LRTQAKCNPNLH 516...
SUDV ...219 EYEIENFGAQHSTTLFKIDNNTFVRLDRPHTPQFLFQLNDTIHLHQQLSNTTGRLIWTLDANINADIGEW 288...505 TKATGKCNPNLH 516...
TAFV ...219 NYVVDNFGTNTTEFLFQVDHLTYVQLEARFTPQFLVLLNETIYSDNRRSNTTGKLIWKINPTVDTSMGEW 288...505 PNTQPKCNPNLH 516...
RESTV ...220 SYEMSNFGGNESNTLFKVDNHTYVQLDRPHTPQFLVQLNETLRRNNRLSNSTGRLTWTLDPKIEPDVGEW 289...506 QNTANKCNPDLY 517...
BOMV ...215 EYETTRFGTNRSAFWFKVDNLTFVQLESRFTPQFLVELNETIYIEGKRSNTTGRLIWQVNSRVDTDVGEW 284...501 EIRGAKCNPNLH 512...

Figure S5.

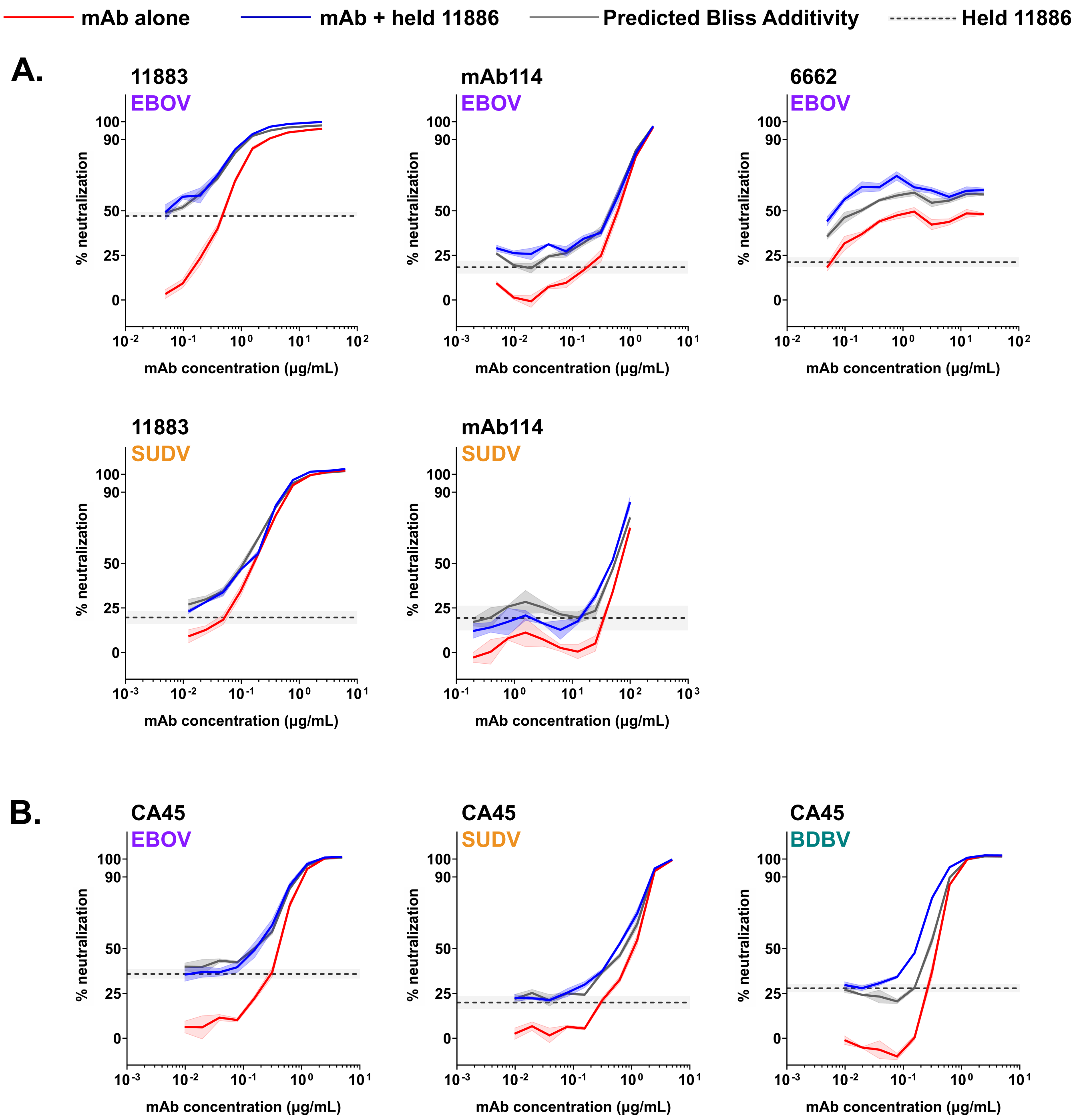

mAb alone
mAb + held 11886
Predicted Bliss Additivity
Held 11886
A.
11883
EBOV
mAb114
EBOV
6662
EBOV
11883
SUDV
mAb114
SUDV
B.
CA45
EBOV
CA45
SUDV
CA45
BDBV
% neutralization
mAb concentration (µg/mL)

Figure S6.

A.

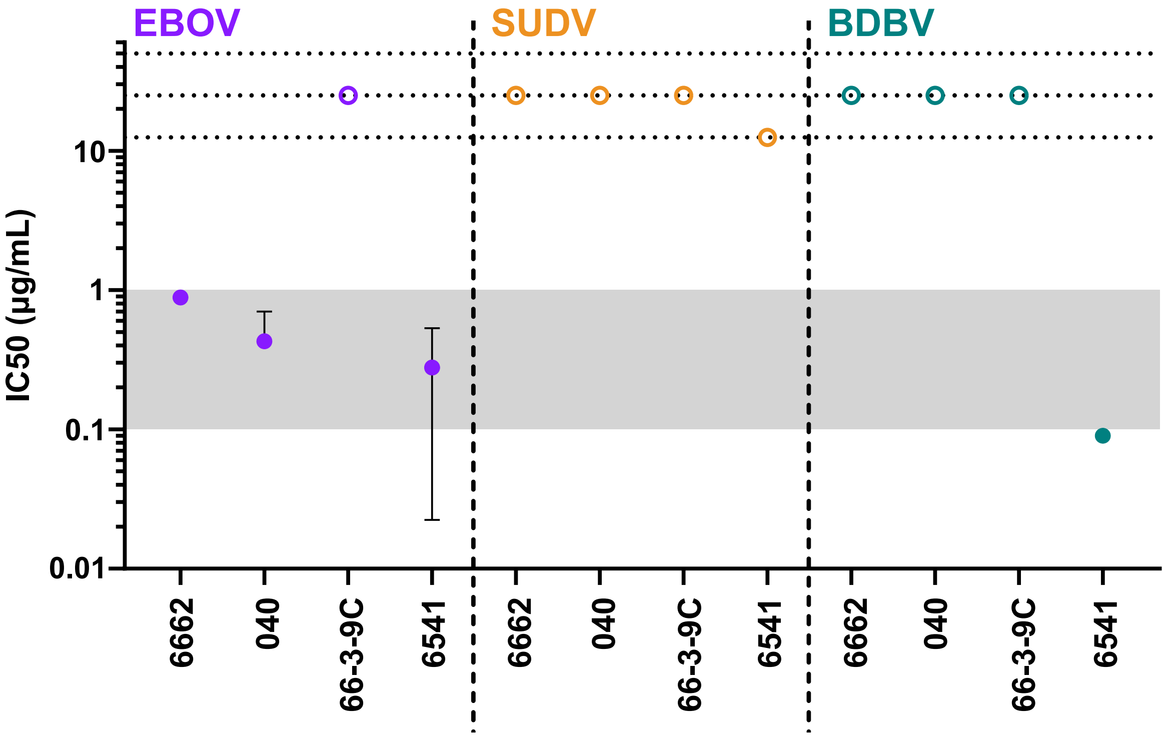

B.

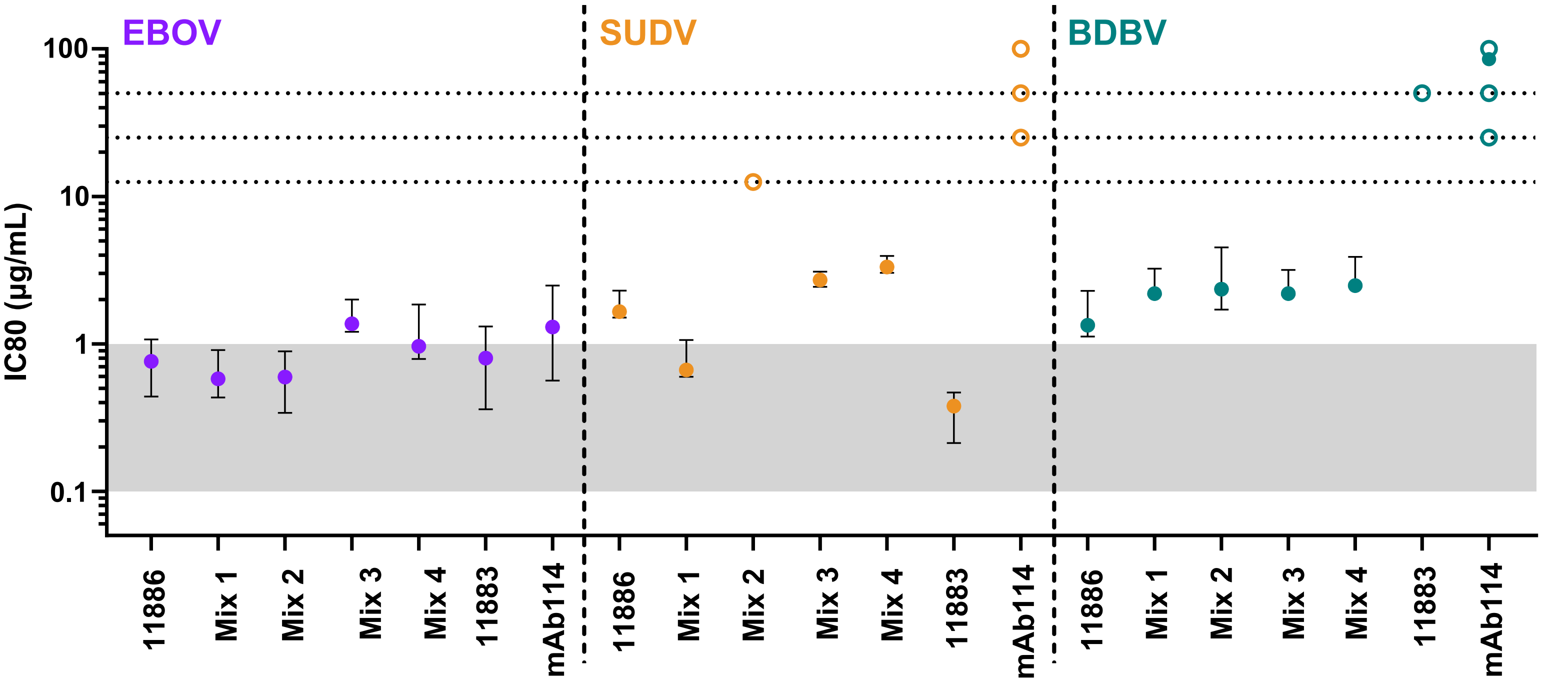

Mix 1	11883 + 66-3-9C + 11886
Mix 2	6662 + 040 + 66-3-9C + 6541
Mix 3	mAb114 + 66-3-9C + 11886
Mix 4	6662 + 040 + 66-3-9C + 11886
